# A retrospective quantitative implementation evaluation of Safer Opioid Prescribing, a Canadian continuing education program

**DOI:** 10.1186/s12909-021-02529-7

**Published:** 2021-02-12

**Authors:** Abhimanyu Sud, Kathleen Doukas, Katherine Hodgson, Justin Hsu, Amber Miatello, Rahim Moineddin, Morag Paton

**Affiliations:** 1 Department of Family & Community Medicine, University of Toronto, Toronto, Canada; 2 Continuing Professional Development, Temerty Faculty of Medicine, University of Toronto, Toronto, Canada; 3 Temerty Faculty of Medicine, University of Toronto, Toronto, Canada

**Keywords:** Continuing education, Opioid, Prescribing, Implementation, Health policy, Complex intervention, Epidemic

## Abstract

**Background:**

Continuing health professions education (CHPE) is an important policy intervention for the opioid epidemic. Besides effectiveness or impact, health policy implementation should be studied to understand how an intervention was delivered within complex environments. Implementation outcomes can be used to help interpret CHPE effects and impacts, help answer questions of “how” and “why” programs work, and inform transferability. We evaluated Safer Opioid Prescribing (SOP), a national CHPE program, using implementation outcomes of reach, dose, fidelity, and participant responsiveness.

**Methods:**

We conducted a retrospective quantitative implementation evaluation of the 2014–2017 cohorts of SOP. To measure reach and dose, we examined participation and completion data. We used Ontario physician demographic data, including regulatory status with respect to controlled substances, to examine relevant trends. To measure fidelity and participant responsiveness, we analyzed participant-provided evaluations of bias, active learning, and relevance to practice. We used descriptive statistics and measures of association for both continuous and categorical variables. We used logistic regression to determine predictors of workshop participation and analysis of covariance to examine variation in satisfaction across different-sized sessions.

**Results:**

Reach: In total, there were 472 unique participants, 84.0% of whom were family physicians. Among Ontario physician participants, 90.0% were family physicians with characteristics representative of province-wide demographics. Dose: Webinar completion rate was 86.2% with no differences in completion based on rurality, gender, or controlled substance prescribing status with medical regulatory authorities. Fidelity and participant responsiveness: Nearly all participants rated the three webinars and workshop as balanced, and each element of SOP was also rated as highly relevant to clinical practice.

**Conclusions:**

This evaluation demonstrates that Safer Opioid Prescribing was implemented as intended. Over a short period and without any external funding, the program reached more than 1% of the Ontario physician workforce. This suggests that the program may be a good model for using virtual CHPE to reach a critical mass of prescribers. This study represents a methodological advance of adapting evaluation methods from health policy and complex interventions for continuing health professions education. Future studies will assess effectiveness and impact on opioid prescribing and utilization within evaluation models of complex interventions.

**Supplementary Information:**

The online version contains supplementary material available at 10.1186/s12909-021-02529-7.

## Introduction

Health policies are often characterized as complex interventions and continuing health professions education (CHPE) programs share many similar distinguishing features: they involve the actions of people, include a complex chain of steps, are embedded in social systems shaped by context, and are dynamic, open systems subject to change [1, 2]. This suggests that such CHPE programs can and should be subjected to the same rigorous evaluations as health policies whenever possible. Studying the implementation of CHPE programs is an important step in not only better understanding if a program was delivered as intended but also in offering further insight into ‘how’ it works to create intended and also unintended effects.

Opioid-related harms such as overdose and death continue to mount throughout high-income North America. In the United States more than 232,000 people died between 1999 and 2018 from opioid overdoses involving prescription opioids, with death rates more than four times higher in 2018 compared to 1999 [3]. Likewise, in Canada the rate of opioid-related deaths has remained persistently elevated with the most recent pre-pandemic rate estimated at 10.7 per 100,000 [4]. While illicit opioids such as fentanyl and its analogues are important drivers of ongoing harms, prescription opioids continue to be important contributors [5]. Similar to the U.S., Canada has recently seen a leveling of national life expectancy for the first time since the Second World War, which has been attributed primarily to opioid-related deaths [6, 7]. Indications are that in many jurisdictions, opioid-related harms have risen significantly in the context of the COVID-19 pandemic [8, 9].

Continuing health professional education (CHPE) without industry involvement has been considered an important rectifying intervention as identified by a variety of national and regional policy documents [10–12], editorialists [13, 14], and national media [15]. The Canadian government has made continued major investments in chronic pain and opioid CHPE as part of its opioid crisis response strategy [16]. This is because education about chronic pain and opioid analgesic prescribing has been considered deficient in terms of both quantity and quality across the medical education continuum [17, 18]. In a recent scoping review of contemporary opioid analgesic CHPE programs [19] we identified that the majority of programs identified the opioid crisis as a motivating factor for developing and delivering their interventions. Likewise, opioid manufacturer promotions couched as educational activities have been prominent drivers of inappropriate prescribing and opioid-related harms [20]. Our review also identified an ongoing influence of opioid prescribing manufacturers in contemporary CHPE programs, an issue which has also been identified in critiques of the Food and Drug Administration’s Opioid Analgesic Risk Evaluation and Mitigation Strategy [21–23].

In a recent systematic review of the effectiveness of interventions to reduce opioid-related harms, Furlan et al. [24] identified CHPE as a promising strategy to improve inappropriate use of opioids and reduce abuse, misuse, and addiction. However, this review did not assess the educational strategies of the CHPE programs that demonstrated these outcomes. At least one systematic review of opioid prescribing interventions has characterized education using an evaluative framework for complex interventions [25]. This review classified CHPE and other prescribing interventions such as prescription drug monitoring programs in terms of implementation, effectiveness and impact outcomes. The study authors, however, did not relate such outcome categorizations back to any kinds of frameworks, conceptual models or theories of educational development, delivery or evaluation.

Moore et al.’s CHPE outcomes framework [26, 27] provides a useful bridge between outcomes for educational programs and complex intervention. The first two levels of participation and satisfaction map clearly to implementation outcomes, the third through fifth levels (knowledge, competence and performance) map to effectiveness outcomes, and the sixth and seventh levels (patient health and population health) can be categorized as typical impact outcomes (Appendix 1). Moore et al. characterize these outcomes as a hierarchy, with the highest quality programs aiming and achieving demonstrable changes in terms of patient or community health outcomes. Evaluations focused on these higher-level outcomes can answer questions such as, “did this program have the intended impacts?” or more bluntly “did this program work?”. However, if program implementation is not studied concurrently with effectiveness and impact outcomes, then such evaluations cannot answer questions of “how” and “why” the programs did or did not have the intended outcomes [28]. Methods of program evaluation which study implementation can aid in opening this so-called “black box problem” [29]. For example, by examining participation and satisfaction, one can learn not only if a program intervention reached its target audience and met its learning objectives, but one can also garner valuable data related to how a program has been implemented in a specific context or how it may be sustained in that context. These data are essential in informing transferability of findings into other contexts [30].

With this framework of opioid prescribing CHPE as a health policy intervention, we aimed to conduct a comprehensive implementation evaluation of a national Canadian program called Safer Opioid Prescribing (SOP). SOP was fully redeveloped from a previous program that has been delivered by the College of Physicians and Surgeons of Ontario (CPSO) to address remediation needs for physicians for whom this medical regulator had identified instances of inappropriate controlled substance prescribing. SOP continued to address this remediation need for the CPSO, as well as medical regulators in other Canadian provinces, but aimed primarily to provide high quality continuing education for health professionals without any regulatory involvement. In this study, we specifically assessed the implementation outcomes of reach, dose, fidelity and participant responsiveness. Study questions, implementation outcomes and corresponding CPD outcome level are summarized in Table 1.

**Table 1:** Mapping study objectives to education and implementation outcomes

Study Questions	Moore et al.’s (26) framework Level	Implementation outcome
1) Who were the participants in the program? Sub-questions: Were family physicians well-represented? Were prescribers from rural and remote communities well-represented? What proportion of participations had involvement with the medical regulator with respect to controlled substance prescribing?	Participation (LEVEL 1)	Reach
2) What was the completion rate of the program? Sub-questions: Which participants were more or less likely to complete the program?	Participation (LEVEL 1)	Dose
3) Did participants note any significant bias in the delivery of the program?	Satisfaction (LEVEL 2)	Fidelity
4) Was adequate time provided for active learning? Sub-questions: How did ratings of active learning change with respect to cohort size?	Satisfaction (LEVEL 2)	Fidelity
5) Was the program relevant to clinical practice? Sub-questions: How did ratings of relevance to practice change with respect to cohort size?	Satisfaction (LEVEL 2)	Participant responsiveness

## Methods

We conducted a quantitative retrospective cohort evaluation of SOP between 2014 and 2017 using registration and participant evaluation data. Ethical approval for this study was granted by the University of Toronto Research Ethics Board.

### Intervention: Safer Opioid Prescribing development and description

Starting in 2012, we developed SOP using Kern’s model for curriculum development [31] with two particular adaptations for CHPE. First, for needs assessment and program planning, we used the PRECEDE-PROCEED model [32, 33], a comprehensive framework for program design, implementation and evaluation that is commonly used in the fields of public health and health promotion. In using this model, we formalized our conception of education as a health policy intervention as has been done elsewhere [34]. Using PRECEDE-PROCEED allowed us to: a) *contextualize* CHPE within the specific circumstances of the Canadian contemporary opioid epidemic and the range of other policy options for addressing it; b) *involve* the target audience for the intervention in program planning; and, c) *conceptualize* and *categorize* specific implementation and effectiveness outcomes during the initial design stages (Table 2; see also Appendix 2 for Program Logic Model).

**Table 2:** Mapping Needs to Program Development

Identified need	➔	How this was addressed in program development
Prescribed opioids were identified as an important contributor to opioid related harms and family physicians were identified as the most common prescribers of opioids [35].	➔	The scientific planning committee included family physicians from a diversity of backgrounds (primary care, chronic pain care, addictions medicine, anesthesia, pharmacology and inner-city medicine).
The opioid epidemic was growing in scale and was linked to the practices of the majority of family physicians [36].	➔	The program targeted family physicians, though it was designed to also be relevant to specialist prescribers as well as other professionals involved in opioid prescribing (e.g. pharmacists). Nurse practitioners were not identified as a primary target at the time of development since they were not eligible to prescribe opioids in our jurisdiction until early 2017.
There was an inequitable distribution of harms, with greater rates of overdoses and deaths from opioids in rural and remote communities – places where there might be less access to practice supports and high quality CHPE programs [37].	➔	The program was to be delivered virtually and in the evenings, outside of typical practice times, to increase accessibility for rural and remote health professionals.
Chronic pain was a major learning priority for family physicians [38, 39] and there were important knowledge gaps with respect to opioid prescribing [40].	➔	SOP content focused on opioid prescribing but was contextualized within models of the management of chronic pain as a complex medical condition.
There was a persistent influence of the pharmaceutical industry on prescribing practices and thus a growing skepticism of opioid educational programs because of possible pharmaceutical industry involvement [20].	➔	Faculty for the program during the study period of interest did not have any history of involvement with opioid or other pharmaceutical manufacturers. The program received no funding from industry for either development or delivery. It was funded entirely by participant registration fees to ensure sustainability. Fees for the program for physician participants were C$450 for the webinars and $650 for the workshops. A reduced rate for non-physician and resident participants was C$150 for the webinars and C$200 for the workshops.
Existing CHPE programs in the field tended to be based on expert opinion rather than the best available evidence, for example, from systematic reviews or clinical practice guidelines.	➔	Foundational documents included a national clinical practice guideline [41] and tool that was developed to support the implementation of the guideline [42].
The provincial medical regulator had an active and substantial influence on opioid prescribing behaviour, which in some cases could be an even stronger driver of prescribing behaviour than certain kinds of educational interventions [43].	➔	Participants in the program were sometimes required or suggested to attend by their medical regulator due to the identification of possible inappropriate controlled substance prescribing; however, program administration and faculty were blinded to participants’ regulatory status.

Our second adaptation to CHPE was to use multiple systematic reviews of continuing medical education (CME) effectiveness to identify and incorporate best practices in education for achieving practice change and improvement in patient outcomes [44–47], including for internet-based CHPE [48]. SOP utilized multiple interventions (13 distinct interventions), was of substantial duration (3–4 months), utilized a blended-learning approach, was interactive, and identified links between clinical practice and serious health outcomes [49]. The program was split into two components – a series of three synchronous evening webinars followed by a one-day in-person workshop to create a flipped classroom (see Appendix 3: Safer Opioid Prescribing Program Components and Description). Besides addressing accessibility, the virtual format also aimed to make the program scalable to reach a large number of participants simultaneously. The webinars were made synchronous and interactive to help create a virtual community of learning [50], which we hypothesized would help normalize a challenging area of practice and also drive higher levels of completion — a known challenge for online learning programs [51, 52].

The first of the three webinars focused on the multimodal management of chronic pain; the second on the details of opioid prescribing (e.g. patient risk assessment, medication selection, initiation and titration); and the third on situations in which prescribing can be more challenging (e.g. with the elderly, in pregnancy, with people living with opioid use disorder). The workshop addressed challenging cases and communication issues, focusing on skills and competencies particularly suited for a live workshop as compared to a synchronous webinar. Webinar participation was a pre-requisite for workshop participation. Each webinar and the workshop had specific pre-work and post-work to prime learning and to facilitate integration into practice, respectively. The program was accredited for a total of 27 credits of learning: 9 credits for the webinars and an additional 18 credits for the workshop.

### Study population

The study population included all SOP participants from January 1, 2014 through June 14, 2017. This study period was chosen because the program had fully launched in its current form by January 2014 and the content of the program was substantially redeveloped after June 2017 based on the release of new Canadian guidelines for opioid prescribing [53]. We included all participants in this study period, regardless of profession, specialty, location, completion status and whether or not they had any identified medical regulator involvement with respect to controlled substance prescribing. We excluded medical residents and trainees, participants for whom there was substantial missing participation data, and participants who participated only in the workshop but not in the webinars.

### Data sources

Participation data was collected from the registration system of Continuing Professional Development at the University of Toronto which administers SOP. Registration data included dates of participation, practice location, profession and specialty. For Ontario physicians, these registration data were linked with gender, graduating medical school and dates of Ontario licensure from the public register of the CPSO. Prior authorization from the CPSO to access these data was obtained. The Ontario Medical Association’s (OMA) Rurality Index of Ontario was used to determine a rurality score based on practice postal code. This Index has been used in other program evaluations as a measure of rurality for Ontario physicians [54]. For data pertaining to the rurality of the Ontario family physician population (as a comparator to our participant group), we combined the OMA-generated RIO score at the Census Sub-Division level [55] with the Ontario Physician Human Resources Data Center’s list of Physician Counts by Census Sub-Division for 2017 [56]. For satisfaction data, we used anonymous program evaluations collected immediately post program.

### Outcome measures

We collected both participation and satisfaction data to assess for the implementation measures of reach, dose, fidelity and participant responsiveness [57]. We defined program reach as the total number of participants in any webinar. We further characterized this outcome with information about their profession, specialty and province of practice. Specifically, for Ontario physician participants, we collected data about gender, graduating medical school (international versus domestic), number of years of practice since Ontario licensure to first participation in SOP, medical specialty and rurality. This aligns with Durlak and DuPre’s [57] definition of reach as, “the percentage of the eligible population who took part in the intervention, and their characteristics” (p 329). Given the known influence of medical regulation on opioid prescribing [43], we also recorded the status of the Ontario physician participants with respect to controlled substance prescribing and the provincial medical regulatory college (CPSO). We defined those who had a public record of controlled substance prescribing restrictions or a public record of a regulatory hearing regarding their controlled substance prescribing as having medical regulatory involvement.

Hewing closely to the definition of dose as “how much of the original program has been delivered” ([57] , pp. 329), we measured program dose using attendance information for each of the webinars and workshop. Attendance at each of the webinars and workshop was coded as a binary attended / did not attend outcome.

Since reducing bias, and specifically bias relating to opioid manufacturer involvement was a key element of the intended program design, we interpreted ratings of program bias as a measure of program fidelity – namely “the extent to which the innovation corresponds to the originally intended program” ([57 ], pp.329). Participants responded “yes” or “no” to the question “Was the presentation balanced and unbiased?”. Likewise, the program was also intentionally designed to directly address identified clinical needs of practicing physicians which centered around appropriate chronic pain management, alongside opioid prescribing. Thus, we interpreted participant-provided relevance to practice ratings as a measure of program fidelity. This was measured using a 5-point Likert scale where 1 is ‘not at all relevant’ and 5 is ‘very relevant’ for the webinars and a 7-point Likert scale where 1 is ‘not at all relevant’ and 7 is ‘very relevant’ for the workshops.

Finally, we used anonymized, participant-provided ratings of adequacy of active learning time as a measure of participant responsiveness, which can be defined as “the degree to which the program stimulates the interest or holds the attention of participants” ([57] , pp. 329). This was measured using a 5-point Likert scale where 1 is ‘none’ and 5 is ‘just enough’ for the webinars and a 7-point Likert scale where 1 is ‘none’ and 7 is ‘just enough’ for the workshops.

These last three measures for fidelity and participant responsiveness were collected anonymously from participants post-intervention and so could not be linked to participant demographics.

### Data analysis

We used descriptive statistics mean, median, standard deviation, minimum, and maximum for continuous measures, and frequency and percentage for categorical measures to describe the sample. We assessed the association between categorical variables using Chi-squared and Fisher’s exact tests. We assessed the association between binary and continuous measures using two sample t-test. We used logistic regression to assess association between participant factors including gender, years in practice, webinar completion, regulatory college status, setting, and international medical graduate (IMG) or Canadian medical graduate (CMG) status and the likelihood of workshop participation. We used the Hosmer–Lemeshow test to assess the goodness of fit for the logistic regression model and the outcomes are reported as odds ratios. We used analysis of covariance to assess for variability in adequacy of active learning time and clinical relevance across different size groups and program types (webinar or workshop). All tests were two-sided and *p* < 0.05 was considered statistically significant. We used the statistical software SAS 9.4 for data manipulation and statistical analysis.

## Results

During this study period, the SOP series of three webinars was offered 11 times. The workshop was offered 8 times as a standalone program in Toronto, Ontario, Canada at a University-affiliated conference centre. The number of participants per webinar ranged from 26 to 74 (*mean* = 47.5). Workshop participation ranged from 14 to 26 (*mean* = 24.2). The response rate for anonymous post webinar evaluations was between 51 to 53% and for post workshop evaluations was 91 to 99%.

### Reach

#### Participant characteristics

There were 517 unique registrants for this program. Participants who only participated in the workshop (*n* = 10) were excluded from this analysis. We combined data for registrants who participated in the program more than once (*n* = 20) into a single record. We excluded 18 medical residents (3.5%) and also excluded an additional 17 registrants (3.3%) due to incomplete registration or participation data.

In total, there were 472 unique participants (Table 3). One hundred and sixty-four (34.7%) participants were female. The large majority (88.1%) were from Ontario while the remainder were from each of the other Canadian provinces but none of the northern territories. There were three participants from a US state adjacent to Ontario. Three hundred and ninety-eight (84.3%) were family physicians which included general practitioners as well as those with focused practices in emergency medicine, addiction medicine, anesthesiology, community medicine, geriatrics, palliative care, occupational medicine and psychotherapy. Fifty-two (11.0%) were other medical specialists with the majority being from emergency medicine and anesthesiology. Twenty-one (4.4%) were other health professionals, including dentists, pharmacists, registered nurses and nurse practitioners, all of whom were from Ontario.

**Table 3:** Program Reach - Participants by Gender and Profession (overall and Ontario)

		All participants (%)	All Ontario participants (%)
Gender	F	164 (34.7)	140 (33.6)
	M	308 (65.2)	276 (66.3)
Profession	Family physician (total)	398 (84.3)	360 (86.5)
	General family medicine	360 (76.3)	331 (79.6)
	Emergency medicine	25 (5.3)	22 (5.3)
	Addiction medicine, Community medicine, Occupational medicine, Palliative care, Anesthesiology, Geriatrics, Psychotherapy, Sports medicine	< 5 each	< 5 each
	Other medical specialist (total)	53 (11.2)	40 (11.1)
	Anesthesiology	14 (3.0)	11 (2.6)
	Emergency Medicine	14 (3.0)	11 (2.6)
	Community Medicine, Endocrinology, Gastroenterology, Hematology, Internal Medicine, Nephrology, Neurology, Occupational medicine, Orthopedic surgery, Pathology, Pediatrics, Psychiatry, Vascular surgery	< 7 each	< 7 each
	Other health professions	22 (4.7)	16 (3.8)
	Nurse practitioners	7 (1.5)	5 (1.2)
	Nursing, Dentistry, Pharmacy, Unknown	< 5 each	< 5 each

#### Ontario physician sample

**Fig. 1:**
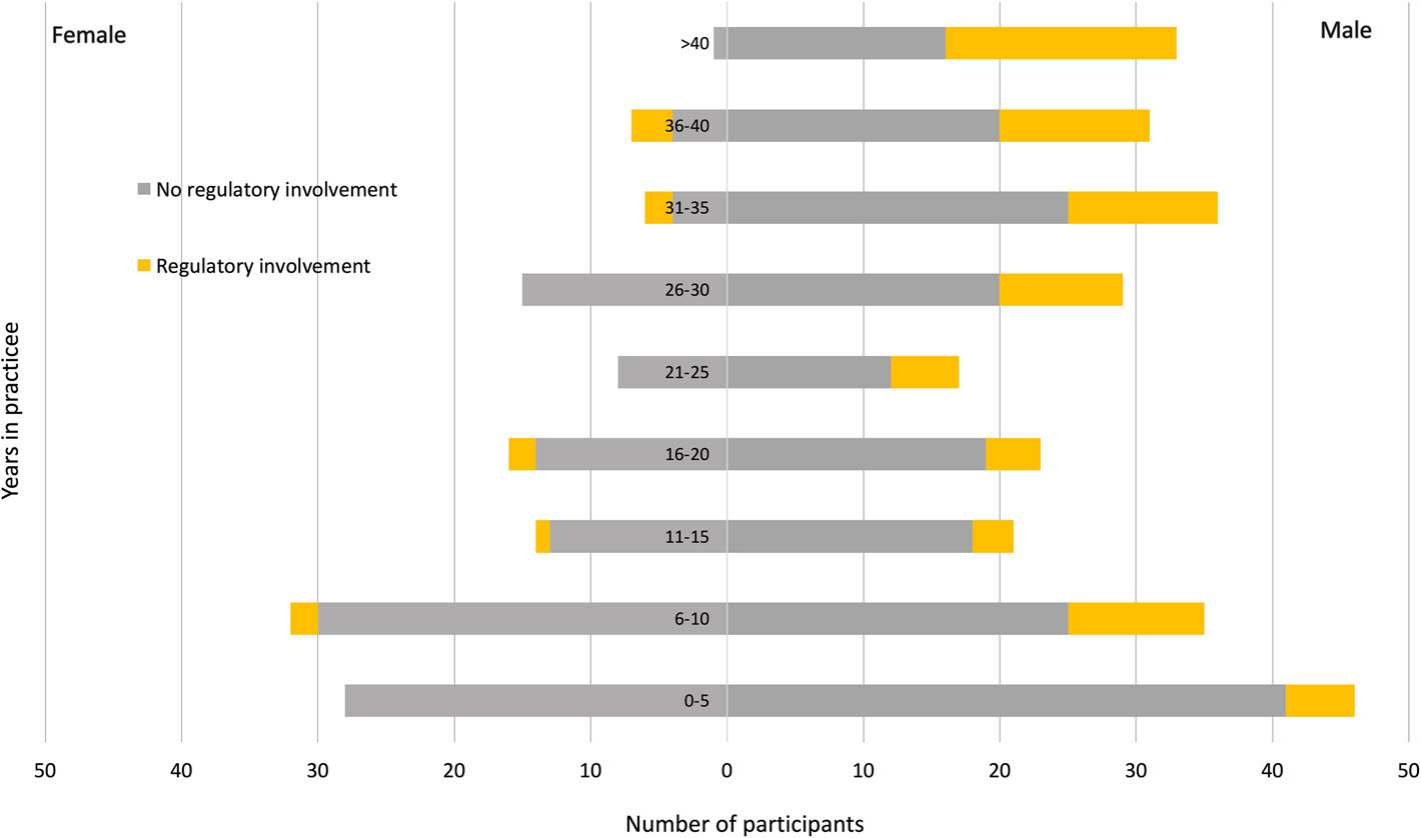
Ontario physician participant distribution by years in practice, gender and regulatory status

Among the Ontario physician participants, family physicians were clearly over-represented in this sample at 90.0% compared to 50.2% of the Ontario physician workforce (*p* < .0001). Two hundred and fifty-nine (64.8%) were Canadian medical graduates, which was significantly lower than the proportion of 70.5% for physicians in the entire province (*p* < .0001). The mean number of years of practice was 19.2 (*SD* = 14.0) and ranged from 0.0 to 50.0. There was a clear bimodal distribution with a peak in the 0–10 year range consisting of nearly even female and male participants and another peak in the 30–35 years in practice range consisting mostly of male participants (Fig. 1).

While the overall sample was less urban than was the Ontario physician workforce, this difference was not significant when the setting distribution was compared by physician specialty. The rural to urban distribution of Ontario family physician and medical specialist participants were both reflective of the distribution of all physicians in the province (not shown).

#### Regulatory college status

We analyzed the Ontario physician participants with respect to their status regarding controlled substance prescribing with the provincial medical regulatory college (Table 4). We found that SOP participants with regulatory involvement were much more likely to be male and to have been in practice for longer. There were no differences in the rural to urban distribution, country of medical school graduation or profession type.

**Table 4:** Reach - Ontario physician participant demographics: all compared to Ontario population, non-CPSO compared to CPSO

		Ontario physician participants, ***n*** = 400 (%)	All Ontario physicians 2017, ***N*** = 32,055 (%)	***p*** value	Ontario physician non-CPSO (%)	Ontario physician CPSO (%)	***p*** value
**Gender**	M	273 (68.2)	19,422 (60.7)	**.002**	198 (62.9)	75 (88.2)	**< .001**
	F	127 (31.8)	12,760 (39.3)		117 (37.1)	10 (11.8)	
**Profession**	Family physician	360 (90.0)	16,088 (50.2)	**< .001**	282 (89.5)	79 (92.9)	.34
	Other specialist	40 (10.0)	15,967 (49.8)		33 (10.5)	6 (7.1)	
**Setting**	Urban	308 (77.2)	25,692 (83.9)	**<.001**	247 (78.4)	61 (71.76)	.45
	Non-major urban	62 (15.5)	3610 (11.8)		47 (14.9)	15 (17.6)	
	Rural	29 (7.3)	1305 (4.3)		20 (6.4)	9 (10.6)	
	Unknown	1 (0.2)	0 (0.0)		1 (0.3)	0 (0.0)	
**Place of medical graduation**	Canada	259 (64.8)	22,614 (70.5)	**< .001**	202 (64.1)	57 (67.1)	.62
	International	141 (35.2)	9437 (29.4)		113 (35.9)	28 (32.9)	
	Unknown	0 (0.0)	4 (0.0)		0 (0.0)	0 (0.0)	
**Mean years in practice (*SD*)**		19.2 (14.0)	N/A	–	17.1 (13.4)	26.5 (14.1)	**<.001**

### Dose

#### Webinar completion

Of the 472 webinar participants, 407 completed all three webinars for a completion rate of 86.2% (Table 5). There was no difference in completion based on gender, rural to urban setting in Ontario, country of graduation, or status with the regulatory college. The completion rate for family physicians (88.4%) and other health professionals (85.7%) was significantly higher than for other medical specialists (69.2%) (*p* = 0.001). Family physicians with a focused practice in emergency medicine (*n* = 25) had a completion rate of 68.0%, which was closer to other medical specialists than to other family physicians (not shown). Likewise, Ontario participants had a significantly higher completion rate of 87.7% compared to the non-Ontario rate of 75.0% (*p* = .009). However, participants from jurisdictions below a two-hour time difference from Ontario (Quebec, Manitoba, Nova Scotia, New Brunswick, Newfoundland and US) had completion rates similar to Ontario at 85.2% (*p* = .753), while those at a two-hour or greater time difference from Ontario (Saskatchewan, Alberta, British Columbia) had a significantly lower completion rate of 65.5% (*p* = .0005).

**Table 5:** Dose - Webinar Completion and Workshop Participation Rates for all Participants

		Webinar completers (%)	***p*** value	Workshop participation (%)	***p*** value
**Overall**		407 (86.2)	–	177 (37.5)	–
**Gender**	Male	266 (86.4)	.907	125 (40.6)	.058
	Female	141 (86.0)		52 (31.7)	
**Setting**	Urban	285 (88.5)	.591	123 (38.3)	.199
	Non-major urban	52 (83.9)		25 (40.3)	
	Rural	27 (87.1)		17 (54.8)	
**Place of medical graduation**	Canada	230 (88.8)	.286	106 (40.9)	.968
	International	120 (85.1)		58 (41.1)	
**Regulatory status**	No CPSO involvement	272 (86.3)	.180	105 (33.3)	**<.0011**
	CPSO involvement	78 (91.8)		59 (69.4)	
**Profession**	Family physician	352 (88.4)	**<.001**	162 (40.7)	**.004**
	Other specialist	36 (69.2)		12 (23.1)	
	Other health profession	18 (85.7)		3 (14.3)	
**Province**	Ontario	365 (87.7)	**.009**	166 (39.9)	**.003**
	All other	42 (75.0)		11 (19.6)	
	< 2-h time difference	24 (85.2)	.753	–	–
	> 2-h time difference	18 (65.5)	**<.001**	**–**	**–**

#### Workshop participation

Of the 472 webinar participants, 177 (37.5%) participated in the workshop. Ontario participants were more likely to participate in the workshop than were participants from other provinces (participation rate 39.9% versus 19.6%, *p* = .003). We conducted a multivariate logistic regression to determine predictors of workshop participation for Ontario physician participants. Webinar completers were 5.1 times more likely to participate in the workshop (*p* < .001; 95% CI = 2.1 to 12.1) than non-completers. Those who had medical regulatory involvement were 4.1 times more likely than those with no involvement to participate in the workshop (*p* < .001, 95% CI = 2.3 to 7.2). Urban practitioners were 2.2 times more likely than rural practitioners to participate in the workshop, but this finding was not statistically significant (*p* = .083, 95% CI = 0.9 to 5.1). There was no difference between urban and non-major urban participants (*p* = .32, 95% CI = 0.6 to 1.9). Gender, years in practice, or country of medical school graduation also did not predict workshop participation.

### Fidelity

Close to 100% of the participants rated the three webinars and workshop as balanced and unbiased (Table 6). Each element of SOP was also rated as highly relevant to clinical practice. An analysis of covariance showed that the effect of group size on relevance to practice when controlling for program type (webinar or workshop) was not significant (*p* = .514).

**Table 6:** Fidelity and Participant responsiveness - Participant Evaluations of Bias, Relevance to Practice, and Adequacy of Active Learning Time

	Webinar 1	Webinar 2	Webinar 3	Workshop
**% reporting balance and no bias**	96.7	99.0	98.4	98.0
**Relevance to practice, mean (SD)**	4.5 (0.2)/ 5	4.5 (0.2)/ 5	4.5 (0.2)/ 5	6.4 (0.1)/ 7
**Adequate time for active learning, mean (SD)**	4.4 (0.3)/ 5	4.4 (0.2)/ 5	4.3 (0.3)/ 5	6.3 (0.3)/ 7

### Participant responsiveness

All elements of the program were rated as having adequate time for active learning (Table 6). An analysis of covariance showed that the effect of group size on adequacy of active learning time when controlling for program type (webinar or workshop) was not significant (*p* = .178).

## Discussion

### Interpretation of results

SOP was designed as a policy intervention for the Canadian opioid crisis, using best practices in CHPE as a means for driving meaningful change at multiple levels of outcomes. This evaluation strongly suggests that through 2014–2017, SOP was delivered as intended along multiple implementation outcomes including reach, dose, fidelity and participant responsiveness.

Family physicians, who are responsible for the majority of long-term and high-dose opioid prescribing, were disproportionately represented in the program. Likewise, participation in the program was representative of the geographic spread of physicians in Ontario, despite the program being delivered virtually from a major urban academic medical centre. This was accomplished in the context of Ontario’s vast geography and known issues of poor access to high-speed internet in rural and remote communities [58]. Overall, physician participants were not reflective of the gender mix of Ontario physicians. However, this was mostly driven by participants with medical regulatory involvement, who very much skewed male and also as having more years in practice. This cohort of participants with medical regulatory involvement is reflective of patterns in Ontario that have been identified with respect to potentially problematic opioid prescribing [35] and also patterns of medical regulatory referral to opioid education programs [43].

The program was rated as highly relevant to practice, which may partly explain the very high engagement and completion rates. The slightly lower completion rates amongst specialist physicians and family physicians with focused practices, such as in emergency medicine, may in part be driven by lower relevance to practice ratings. It should be noted, however, that completion rates amongst these groups was still very high compared to internet-delivered CHPE norms [59–61]. Another reason for lower completion rates might also be less predictable clinical schedules for certain specialists. Importantly, medical regulator involvement was not an important driver of webinar completion.

Out of province completion rates were also high at 75.0%, though not as high as Ontario participants. This may be driven by lower relevance to practice as some of the program content is focused on epidemiological and practice issues specific to Ontario. However, a more practical reason for this difference may be because of time zone differences making it challenging for working professionals to participate in the webinars.

While not formally assessed as part of this implementation evaluation, scalability of the program [62] is suggested by the variable size of the webinars, with the largest including 74 simultaneous learners, and no systematic variation in time for active learning or relevance to practice based on webinar size. This suggests that fidelity to the program was maintained even with large numbers of participants. Overall, over a short period, the program was able to reach more than 1% of the 32,055 strong Ontario physician workforce. The demonstrated geographic reach of the program and the potential for scalability suggest that this program may be good model for reaching a critical mass of prescribers to drive population level changes in opioid utilization.

### Implications

As CHPE evaluation has moved increasingly towards outcome-based approaches [28] with a preference for “higher-level” outcomes such as patient-level and population-level outcomes, there has been a related discounting of implementation outcomes such as participation and satisfaction [63]. Indeed Moore et al.’s updated framework [27] refers to three different kinds of assessment, namely summative, performance and impact. Importantly, however, this framework ignores assessment of implementation. This may be because educational interventions are not commonly conceptualized as complex interventions that are delivered in complex and dynamic health system and policy contexts – all of which can affect program delivery and structure (implementation) and thus program effects. Thus, rigorous implementation evaluations can be used to determine how the program was actually carried out and whether it was carried out as intended. While implementation of programs as intended is no guarantee of effectiveness, these data are key to then informing subsequent effectiveness and impact evaluations and also to assessing program theory. Having conducted this implementation evaluation, further evaluation of SOP is now called for to assess effectiveness and impact.

To our knowledge, this study is one of few examples of an opioid prescribing CHPE evaluation that has formally assessed implementation outcomes using an evaluation framework for complex interventions together with a CHPE outcome model. Barth et al. [64] describe the use of the Medical Research Council complex intervention framework to develop and evaluate, in a step-wise manner, an academic detailing intervention to improve use of a prescription drug monitoring program (PDMP). A subsequent study evaluated physician self-reports of PDMP utilization – namely a performance (education) or effectiveness (complex intervention) outcome [65]. Other opioid prescribing CHPE programs have assessed implementation measures of participation and satisfaction [66–70] but have not directly related these measures to program theory, nor have they used these implementation measures to then inform effectiveness or impact outcomes [19].

Overall this implementation evaluation adds further support to the feasibility of delivering multicomponent CHPE programs virtually to increase reach, scalability and thus potentially effectiveness and impact [71–73]. This is particularly relevant for population health problems directly linked to clinical practices of which the opioid epidemic is a pertinent example. Likewise, in the context of the COVID-19 pandemic and related physical-distancing measures, this evaluation provides additional confidence that virtual programs that adhere to evidence-based CHPE practices can provide good models to meet the ongoing learning needs of clinicians, including in complex areas of practice.

### Limitations

There are several important limitations to this study. First, this study was of a single program and was conducted retrospectively using data that were collected for both evaluative purposes but also for administrative purposes, such as for tracking participation for accreditation reporting. We did have to exclude 17 participants (3.2%) due to incomplete participation data. This was a small enough number that it was unlikely to significantly bias results. Likewise, demographic data was for the most part complete. We could not, for example, determine rurality for only two of the 400 Ontario physician participants. Also, it is important to note that the evaluative data collected (e.g. relevance to practice and amount of interactivity) was defined prior to the delivery of the program and did reflect attempts to assess underlying program theory. The second data limitation relates to the anonymous nature of the evaluative data. These were kept anonymous as per norms in CHPE to allow participants to freely share their evaluative assessments. However, this did not allow us to link evaluative statements to particular participants and then analyze by demographic factors. This could be rectified in future evaluations by using, for example, a linking identifier that blinds the scientific planning committee to the identity of participants but allows evaluators to link evaluations to the demographic characteristics of de-identified program participants. Likewise, the webinar evaluation data response rates were moderate at 51% which would introduce an unknown bias to these data. Since these responses were anonymous, it is not possible to further assess the nature of this possible bias. However, the consistency of these evaluative responses between the webinars and the consistency of the responses with the workshop data which had an excellent response rate provides confidence that these data are reflective of the entire participant population. Third, the available data did not allow for a direct inquiry into the posited mechanism for change, namely that the SOP structure facilitates the creation of a virtual community of learning and practice. Qualitative inquiry using interviews or focus groups of program participants and facilitators would be well suited to better assess this aspect of the program. As noted above, this evaluation did not study patient- or population-level outcomes, which will be a priority for future study.

## Conclusions

This evaluation demonstrates that Safer Opioid Prescribing was implemented as intended. Over a short period and without any external funding, the program reached more than 1% of the Ontario physician workforce. This suggests that Safer Opioid Prescribing may be a good model for using virtual continuing health professions education to reach a critical mass of prescribers. This study represents a methodological advance of adapting evaluation methods from health policy and complex interventions for continuing health professions education. This implementation evaluation will be used to inform further effectiveness and impact evaluations of Safer Opioid Prescribing.

## Supplementary Information

**Additional file 1.**

**Additional file 2.**

**Additional file 3.**

## Data Availability

With the exception of data on physician regulatory status, datasets used and/or analyzed during the current study are available from the corresponding author on reasonable request. The data that support the findings of physician regulatory status of this study are available from the College of Physicians and Surgeons of Ontario but restrictions apply to our further circulation of these data, which were used with their permission for the current study.

## References

[1] Pawson R, Greenhalgh T, Harvey G, Walshe K (2004). Realist synthesis: an introduction.

[2] Sridharan S, Nakaima A (2011). Ten steps to making evaluation matter. Eval Program Plann.

[3] National Center for Health Statistics Centers for Disease Control and Prevention (2020). Wide-ranging online data for epidemiologic research (WONDER).

[4] PHAo C, Special Advisory Committee on the Epidemic of Opioid Overdoses (2020). Opioid-related Harms in Canada.

[5] Gomes T, Khuu W, Martins D, Tadrous M, Mamdani MM, Paterson JM, et al. Contributions of prescribed and non-prescribed opioids to opioid related deaths: population based cohort study in Ontario, Canada. BMJ. 2018;362:k3207. 10.1136/bmj.k3207 PMC6113771 30158106

[6] Statistics Canada (2017). Changes in life expectancy by selected causes of death.

[7] Woolf SH, Schoomaker H (2019). Life expectancy and mortality rates in the United States, 1959-2017. JAMA..

[8] Ontario Drug Policy Research Network, Office of the Chief Coroner for Ontario/Ontario Forensic Pathology Service, Ontario Agency for Health Protection and Promotion (Public Health Ontario), Centre on Drug Policy Evaluation (2020). Preliminary Patterns in Circumstances Surrounding Opioid-Related Deaths in Ontario during the COVID-19 Pandemic.

[9] Government of Alberta (2020). Alberta COVID-19 Opioid Response Surveillance Report: Q2 2020.

[10] Kahn N, Chappell K, Regnier K, Travlos DV, Auth D (2019). A collaboration between government and the continuing education community tackles the opioid crisis: lessons learned and future opportunities. J Contin Educ Heal Prof.

[11] Canadian Centre on Substance Use and Addiction. Joint Statement of Action to Address the Opioid Crisis: A Collective Response. Annual Report 2016-2017. Ottawa: Canadian Centre on Substance Use and Addiction; 2017.

[12] Alford DP (2016). Opioid prescribing for chronic pain—achieving the right balance through education. N Engl J Med.

[13] Mertl S. Doctors need education on prescribing opioids. CMAJ. 2016;188(14):1003. 10.1503/cmaj.109-5322. 10.1503/cmaj.109-5322 PMC5047814 27601601

[14] Motameni AT (2018). Opioids: a need for better education and a call to action. Ann Surg.

[15] Webster F, Rice K, Sud A (2020). A critical content analysis of media reporting on opioids: the social construction of an epidemic. Soc Sci Med.

[16] Canada H. Health Canada's substance use and addictions program: contribution funding; 2020. https://www.canada.ca/en/health-canada/services/substance-use/canadian-drugs-substances-strategy/funding/substance-use-addictions-program.html#wb-auto-4.

[17] Institute of Medicine (2011). Relieving pain in America: a blueprint for transforming prevention, care, education, and research.

[18] Young J, Donahue M, Farquhar M, Simpson C, Rocker G. Using opioids to treat dyspnea in advanced COPD. Attitudes and experiences of family physicians and respiratory therapists. Can Fam Physician. 2012;58(7):e401–7. PMID: 22798476; PMCID: PMC3395547. PMC3395547 22798476

[19] Sud A, Salamanca-Buentello F, Molska G. Evaluations of continuing health provider education for opioid prescribing: a systematic scoping review. Acad Med. In Press. 10.1097/ACM.0000000000004186 PMC8781229 34074902

[20] Van Zee A (2009). The promotion and marketing of oxycontin: commercial triumph, public health tragedy. Am J Public Health.

[21] Food US, Administration D. Opioid analgesic risk evaluation and mitigation strategy (REMS); 2018. https://www.fda.gov/drugs/information-drug-class/opioid-analgesic-risk-evaluation-and-mitigation-strategy-rems.

[22] Heyward J, Olson L, Sharfstein JM, Stuart EA, Lurie P, Alexander GC (2020). Evaluation of the extended-release/long-acting opioid prescribing risk evaluation and mitigation strategy program by the US Food and Drug Administration: a review. JAMA Intern Med.

[23] Lurie J. Mother Jones [Internet] April 27, 2018. [2020-11-25 18,10:50]. Available from: https://www.motherjones.com/politics/2018/04/doctors-are-required-to-receive-opioid-training-big-pharma-funds-it-what-could-go-wrong/.

[24] Furlan AD, Carnide N, Irvin E, Van Eerd D, Munhall C, Kim J (2018). A systematic review of strategies to improve appropriate use of opioids and to reduce opioid use disorder and deaths from prescription opioids. Can J Pain.

[25] Moride Y, Lemieux-Uresandi D, Castillon G, de Moura CS, Pilote L, Faure M (2019). A systematic review of interventions and programs targeting appropriate prescribing of opioids. Pain Physician.

[26] Moore DE, Green JS, Gallis HA (2009). Achieving desired results and improved outcomes: integrating planning and assessment throughout learning activities. J Contin Educ Heal Prof.

[27] Moore DE, Chappell K, Sherman L, Vinayaga-Pavan M (2018). A conceptual framework for planning and assessing learning in continuing education activities designed for clinicians in one profession and/or clinical teams. Med Teach.

[28] Haji F, Morin M-P, Parker K (2013). Rethinking programme evaluation in health professions education: beyond ‘did it work?’: Rethinking health professions programme evaluation. Med Educ.

[29] Astbury B, Leeuw FL (2010). Unpacking black boxes: mechanisms and theory building in evaluation. Am J Eval.

[30] Damschroder L, Aron D, Keith R, SR K, Alexander J, Lowery J (2009). Fostering implementation of health services research findings into practice: a consolidated framework for advancing implementation science. Implement Sci.

[31] Kern D, Thomas P, Hughes M (2009). Curriculum development for medical education: a six-step approach.

[32] Gielen AC, McDonald EM, Gary TL, Bone LR, Glanz K, Rimer BK, Viswanath K (2008). Using the precede-proceed model to apply health behavior theories. Health behavior and health education: theory, research, and practice.

[33] Green L, Kreuter M (1999). The precede–proceed model. Health promotion planning: an educational approach 3rd ed Mountain View (CA): Mayfield Publishing Company.

[34] White MI (2003). Toward an evidence-informed, theory-driven model for continuing medical education: University of British Columbia.

[35] Dhalla IA, Mamdani MM, Gomes T, Juurlink DN (2011). Clustering of opioid prescribing and opioid-related mortality among family physicians in Ontario. Can Fam Physician.

[36] Dhalla IA, Mamdani MM, Sivilotti ML, Kopp A, Qureshi O, Juurlink DN (2009). Prescribing of opioid analgesics and related mortality before and after the introduction of long-acting oxycodone. CMAJ..

[37] Gomes T, Juurlink D, Moineddin R, Gozdyra P, Dhalla I, Paterson M (2011). Geographical variation in opioid prescribing and opioid-related mortality in Ontario. Healthc Q.

[38] Leverence RR, Williams RL, Potter M, Fernald D, Unverzagt M, Pace W (2011). Chronic non-cancer pain: a Siren for primary care – a report from the PRImary care MultiEthnic network (PRIME net). J Am Board Fam Med.

[39] Johnson M, Collett B, Castro-Lopes JM (2013). The challenges of pain management in primary care: a pan-European survey. J Pain Res.

[40] Allen MJM, Asbridge MM, Macdougall PC, Furlan AD, Tugalev O (2013). Self-reported practices in opioid management of chronic noncancer pain: a survey of Canadian family physicians. Pain Res Manag.

[41] Furlan AD, Reardon R, Weppler C, National Opioid Use Guideline G (2010). Opioids for chronic noncancer pain: a new Canadian practice guideline. CMAJ.

[42] Michael G (2020). DeGroote National Pain Centre MU. Opioid Manager.

[43] Kahan M, Gomes T, Juurlink DN, Manno M, Wilson L, Mailis-Gagnon A (2013). Effect of a course-based intervention and effect of medical regulation on physicians’ opioid prescribing. Can Fam Physician.

[44] Marinopoulos SS, Dorman T, Ratanawongsa N, Wilson LM, Ashar BH, Magaziner JL (2007). Effectiveness of continuing medical education. Database of Abstracts of Reviews of Effects (DARE): Quality-assessed Reviews [Internet]: Centre for Reviews and Dissemination (UK).

[45] Cervero RM, Gaines JK. Effectiveness of continuing medical education: updated synthesis of systematic reviews. Accredit Counc Contin Med Educ. 2014:1–19.

[46] Bloom BS (2005). Effects of continuing medical education on improving physician clinical care and patient health: a review of systematic reviews. Int J Technol Assess Health Care.

[47] Davis D, O'Brien MAT, Freemantle N, Wolf FM, Mazmanian P, Taylor-Vaisey A (1999). Impact of formal continuing medical education: do conferences, workshops, rounds, and other traditional continuing education activities change physician behavior or health care outcomes?. JAMA..

[48] Cook DA, Levinson AJ, Garside S, Dupras DM, Erwin PJ, Montori VM. Instructional Design Variations in Internet-Based Learning for Health Professions Education: A Systematic Review and Meta-Analysis. Acad Med. 2010;85(5):909–22. 10.1097/ACM.0b013e3181d6c319 20520049

[49] Forsetlund L, Bjørndal A, Rashidian A, Jamtvedt G, O'Brien MA, Wolf F (2009). Continuing education meetings and workshops: effects on professional practice and health care outcomes. Cochrane Database Syst Rev.

[50] Wong G, Greenhalgh T, Pawson R (2010). Internet-based medical education: a realist review of what works, for whom and in what circumstances. BMC Med Educ.

[51] Scott KM, Baur L, Barrett J (2017). Evidence-based principles for using technology-enhanced learning in the continuing professional development of health professionals. J Contin Educ Heal Prof.

[52] Goldberg LR, Bell E, King C, O’Mara C, McInerney F, Robinson A (2015). Relationship between participants’ level of education and engagement in their completion of the understanding dementia massive open online course. BMC Med Educ.

[53] Busse JW, Craigie S, Juurlink DN, Buckley DN, Wang L, Couban RJ (2017). Guideline for opioid therapy and chronic noncancer pain. Can Med Assoc J.

[54] Kiran T, Moineddin R, Kopp A, Frymire E, Glazier RH (2018). Emergency department use and enrollment in a medical home providing after-hours care. Ann Fam Med.

[55] Kralj B (2009). Measuring rurality—RIO2008_BASIC: methodology and results: OMA economics department. Toronto, Canada.

[56] Ontario Physician Human Resources Data Centre (2018). County and Census Subdivision Report - Active Physicians in Ontario by County and Census Subdivision (CSD) in 2017.

[57] Durlak JA, DuPre EP (2008). Implementation matters: a review of research on the influence of implementation on program outcomes and the factors affecting implementation. Am J Community Psychol.

[58] Haight M, Quan-Haase A, Corbett BA (2014). Revisiting the digital divide in Canada: the impact of demographic factors on access to the internet, level of online activity, and social networking site usage. Inf Commun Soc.

[59] Lew EK, Nordquist EK (2016). Asynchronous learning: student utilization out of sync with their preference. Med Educ Online.

[60] Margolis A, Gonzalez-Martinez F, Noboa O, Abbud-Filho M, Lorier L, Nin M, et al. Online Continuing Medical Education for the Latin American Nephrology Community. Stud Health Technol Inform. 2015;216:372–5. PMID: 26262074. 26262074

[61] Kiwanuka J, Ttendo S, Eromo E, Joseph S, Duan M, Haastrup A (2015). Synchronous distance anesthesia education by internet videoconference between Uganda and the United States. J Clin Anesth.

[62] Milat AJ, Bauman A, Redman S (2015). Narrative review of models and success factors for scaling up public health interventions. Implement Sci.

[63] Tian J, Atkinson NL, Portnoy B, Gold RS (2007). A systematic review of evaluation in formal continuing medical education. J Contin Educ Heal Prof.

[64] Barth KS, Ball S, Adams RS, Nikitin R, Wooten NR, Qureshi ZP (2017). Development and feasibility of an academic detailing intervention to improve prescription drug monitoring program use among physicians. J Contin Educ Heal Prof.

[65] Larson MJ, Browne C, Nikitin RV, Wooten NR, Ball S, Adams RS (2018). Physicians report adopting safer opioid prescribing behaviors after academic detailing intervention. Subst Abus.

[66] Donovan AK, Wood GJ, Rubio DM, Day HD, Spagnoletti CL (2016). Faculty communication knowledge, attitudes, and skills around chronic non-malignant pain improve with online training. Pain Med.

[67] Allen M, Macleod T, Zwicker B, Chiarot M, Critchley C (2011). Interprofessional education in chronic non-cancer pain. J Interprof Care.

[68] Katzman JG, Fore C, Bhatt S, Greenberg N, Griffin Salvador J, Comerci GC (2016). Evaluation of American Indian health service training in pain management and opioid substance use disorder. Am J Public Health.

[69] Palmer E, Hart S, Freeman PR (2017). Development and delivery of a pharmacist training program to increase naloxone access in Kentucky. J Am Pharm Assoc.

[70] Sanchez-Ramirez DC, Polimeni C (2019). Knowledge and implementation of current opioids guideline among healthcare providers in Manitoba. J Opioid Manage.

[71] Lam-Antoniades M, Ratnapalan S, Tait G (2009). Electronic continuing education in the health professions: an update on evidence from RCTs. J Contin Educ Heal Prof.

[72] Wutoh R, Boren SA, Balas EA (2004). eLearning: a review of internet-based continuing medical education. J Contin Educ Heal Prof.

[73] Cook DA, Levinson AJ, Garside S, Dupras DM, Erwin PJ, Montori VM (2008). Internet-based learning in the health professions: a meta-analysis. JAMA..

