# Supplementary material for: A retrospective quantitative implementation evaluation of Safer Opioid Prescribing, a Canadian continuing education program
Source: BMC Med Educ. 2021 Feb 12;21:101. doi: 10.1186/s12909-021-02529-7 (PMC7880212; doi:10.1186/s12909-021-02529-7)
Supplement: Supplementary file 1 — Additional file 1. [file 12909_2021_2529_MOESM1_ESM.docx]

Appendix 1: Mapping education outcomes to complex intervention outcomes

| **Moore et al.’s CHPE outcome levels** | **Description** | **Complex intervention outcome evaluation type** |
| --- | --- | --- |
| **Participation**  **(LEVEL 1)** | The number of physicians and other who participated in the CME activity | Implementation |
| **Satisfaction**  **(LEVEL 2)** | The degree to which the expectations of the participants about the setting and delivery of the CME activity were met |  |
| **Learning: Declarative knowledge**  **(LEVEL 3A)** | The degree to which participants state *what* the CME activity intended them to know | Effectiveness |
| **Learning: Procedural knowledge**  **(LEVEL 3B)** | The degree to which participants state *how* to do what the CME activity intended them to know how to do |  |
| **Learning: Competence**  **(LEVEL 4)** | The degree to which participants show in an educational setting how to do what the CME activity intended them to be able to do |  |
| **Performance**  **(LEVEL 5)** | The degree to which participants *do* what the CME activity intended them to be able to do in their practices |  |
| **Patient health**  **(LEVEL 6)** | The degree to which the health status of patients improves due to changes in the practice behavior of participants | Impact |
| **Population health**  **(LEVEL 7)** | The degree to which the health status of a community of patients changes due to changes in the practice behavior of participants |  |
