# Supplementary figures and images for: A retrospective quantitative implementation evaluation of Safer Opioid Prescribing, a Canadian continuing education program
Source: BMC Med Educ. 2021 Feb 12;21:101. doi: 10.1186/s12909-021-02529-7 (PMC7880212; doi:10.1186/s12909-021-02529-7)

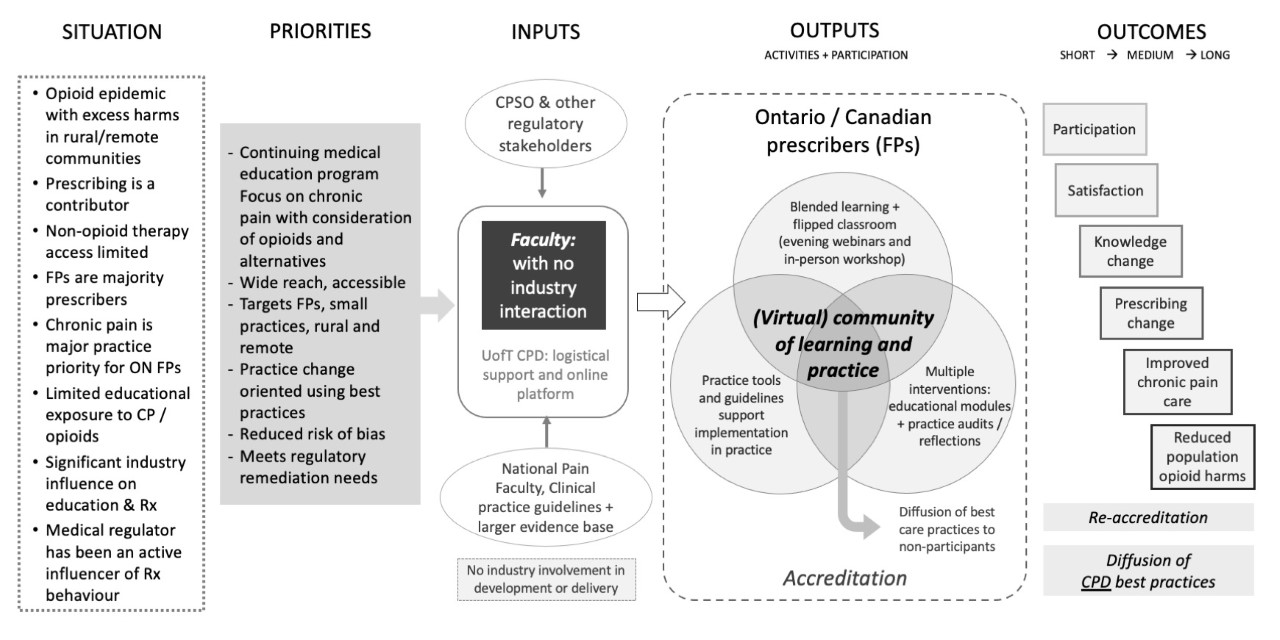

Supplement: Supplementary file 2 — Additional file 2. [file 12909_2021_2529_MOESM2_ESM.jpg]
