# Supplementary material for: A retrospective quantitative implementation evaluation of Safer Opioid Prescribing, a Canadian continuing education program
Source: BMC Med Educ. 2021 Feb 12;21:101. doi: 10.1186/s12909-021-02529-7 (PMC7880212; doi:10.1186/s12909-021-02529-7)
Supplement: Supplementary file 3 — Additional file 3. [file 12909_2021_2529_MOESM3_ESM.docx]

Appendix 3: Safer Opioid Prescribing Program Components and Description

|  | **Webinar 1:**  **Assessing Complex Chronic Pain** | **Webinar 2:**  **Prescribing Opioids for Chronic Pain** | **Webinar 3:**  **Addressing Opioid Challenges and Addiction** | **Workshop:**  **Challenging Cases in Opioid Use and Misuse** |
| --- | --- | --- | --- | --- |
| **PREPARATORY Work** | 1) Review “Is the WHO Analgesic ladder still valid?” and “Neuropathic pain: a practical guide for the clinician”    2) Complete brief practice audit of chronic pain management and opioid prescribing | 1) Review Canadian Guideline for Safe and Effective Use of Opioids for Chronic Non-cancer Pain    2) Complete practice assessment based on first 14 recommendations | 1) Review Canadian Guideline for Safe and Effective Use of Opioids for Chronic Non-cancer Pain    2) Complete practice assessment based on final 10 recommendations | 1) Review “Definitions related to aberrant drug-related behaviour: Is there correct terminology?”    2) Submit summary of a Challenging Case from practice |
| **Examples of active learning and outcomes measures DURING program** | Share practice experience and make a clinical decision: e,g. classify chronic pain versus chronic pain disorder | Calculate patients’ opioid risk score,  calculate total daily dose in morphine equivalence | Share practice experience and make a clinical decision: differentiate addiction, tolerance, and withdrawal | Case study: calculate MEQ  Role play: complete observer checklist |
| **POST PROGRAM practice application exercises** | Report on use of Brief Pain Inventory with 5 patients: assess pain score and functional impairment | Report on use of Opioid Manager with 5 patients: calculate opioid risk and morphine equivalent doses | Submit draft treatment agreement, fax prescription to pharmacy, and consultation request | Practice assessment of skills using the 24 recommendations from clinical practice guidelines |
| **Program spanning methods** | Web-based synchronous webinar distance learning  Virtual learning community | | | Case-based small group interactive workshop |
|  | Blended learning | | | Flipped classroom |
